# Predicting CTS Diagnosis and Prognosis Based on Machine Learning Techniques

**DOI:** 10.3390/diagnostics13030492

**Published:** 2023-01-29

**Authors:** Marwa Elseddik, Reham R. Mostafa, Ahmed Elashry, Nora El-Rashidy, Shaker El-Sappagh, Shimaa Elgamal, Ahmed Aboelfetouh, Hazem El-Bakry

**Affiliations:** 1 Department of the Robotics and Internet Machines, Faculty of Artificial Intelligence, Kafrelsheikh University, Kafr El Sheikh 33516, Egypt; 2 Department of Information Systems, Faculty of Computers and Information, Mansoura University, Mansoura 35516, Egypt; 3 Department of Information Systems, Faculty of Computers and Information, Kafrelsheiksh University, Kafr El Sheikh 33516, Egypt; 4 Department of Machine Learning and Information Retrieval, Faculty of Artificial Intelligence, Kafrelsheiksh University, Kafr El Sheikh 33516, Egypt; 5 Faculty of Computer Science and Engineering, Galala University, Suez 43511, Egypt; 6 Information Systems Department, Faculty of Computers and Artificial Intelligence, Benha University, Banha 13518, Egypt; 7 Department of Neuropsychiatry, Faculty of Medicine, Kafrelsheiksh University, Kafr El Sheikh 33516, Egypt; 8 Delta Higher Institute for Management and Accounting Information Systems, Mansoura 35511, Egypt

**Keywords:** carpal tunnel syndrome (CTS), machine learning (ML), ultrasonography (US), nerve condition studies (NCS), Boston Carpal Tunnel Syndrome Questionnaire (BCTQ)

## Abstract

Carpal tunnel syndrome (CTS) is a clinical disease that occurs due to compression of the median nerve in the carpal tunnel. The determination of the severity of carpal tunnel syndrome is essential to provide appropriate therapeutic interventions. Machine learning (ML)-based modeling can be used to classify diseases, make decisions, and create new therapeutic interventions. It is also used in medical research to implement predictive models. However, despite the growth in medical research based on ML and Deep Learning (DL), CTS research is still relatively scarce. While a few studies have developed models to predict diagnosis of CTS, no ML model has been presented to classify the severity of CTS based on comprehensive clinical data. Therefore, this study developed new classification models for determining CTS severity using ML algorithms. This study included 80 patients with other diseases that have an overlap in symptoms with CTS, such as cervical radiculopathysasas, de quervian tendinopathy, and peripheral neuropathy, and 80 CTS patients who underwent ultrasonography (US)-guided median nerve hydrodissection. CTS severity was classified into mild, moderate, and severe grades. In our study, we aggregated the data from CTS patients and patients with other diseases that have an overlap in symptoms with CTS, such as cervical radiculopathysasas, de quervian tendinopathy, and peripheral neuropathy. The dataset was randomly split into training and test data, at 70% and 30%, respectively. The proposed model achieved promising results of 0.955%, 0.963%, and 0.919% in terms of classification accuracy, precision, and recall, respectively. In addition, we developed a machine learning model that predicts the probability of a patient improving after the hydro-dissection injection process based on the aggregated data after three different months (one, three, and six). The proposed model achieved accuracy after six months of 0.912%, after three months of 0.901%, and after one month 0.877%. The overall performance for predicting the prognosis after six months outperforms the prediction after one and three months. We utilized statistics tests (significance test, Spearman’s correlation test, and two-way ANOVA test) to determine the effect of injection process in CTS treatment. Our data-driven decision support tools can be used to help determine which patients to operate on in order to avoid the associated risks and expenses of surgery.

## 1. Introduction

### 1.1. Overview

Carpal tunnel consists of transverse carpal ligaments and carpal bones. The tunnel is comprised of nine flexor tendons and the median nerve. CTS is the most common entrapment neuropathy and involves pressuring of the median nerve within the carpal tunnel, depending on the severity and duration of neural compression [1]. The impact of carpal tunnel syndrome on productivity, function, and quality of life, as well as the significant costs associated with its management, are associated with a significant socioeconomic burden [2]. The prevalence and severity of CTS increase with age, and women are three times more likely to have CTS than men. Others risk factors include a family history of CTS and a personal history of diabetes, obesity, hypothyroidism, pregnancy, and rheumatoid arthritis. The risk of CTS is also significantly increased by work-related activities that require a high degree of repetition and force or using hand-operated vibratory tools [3,4,5,6].

### 1.2. Problem Statement

The diagnosis of CTS requires listening to the patient’s description of the characteristic timing and distribution of the symptoms and examining the hands to find obvious signs. Electrophysiological studies (EPS) are among the para-clinical diagnostic techniques that are most effective for identifying CTS. They are used by clinicians to group patients based on the severity of their conditions and the need for post-operative care. While NCS has been the gold standard for the diagnosis of CTS, there are still some limitations, because some patients with clinically diagnosed CTS show normal findings [7,8,9]. False-negative results can still happen because tests focus primarily on large, myelinated fibers rather than the small fibers that are responsible for pain. Therefore, clinical history, physical examination, and electrophysiological studies are used to make diagnoses [10]. Imaging techniques can reveal information about the median nerve’s morphology and any local compressive causes. A growing number of studies use ultrasound to evaluate cross-sectional area (CSA) and median nerve compression at the carpal tunnel. It is currently the most accepted method for diagnosing CTS [11] and is economical compared to other imaging methods, such as magnetic resonance imaging (MRI) [12,13,14,15].

A scoring tool has been developed to check for CTS in asymptomatic people, but the claims that the symptoms are only potential ones are called into question. The Michigan Hand Outcomes Questionnaire (MHQ) and the Boston Carpal Tunnel Questionnaire (BCTQ) are two other questionnaires that have been developed to quantify CTS symptoms based on patient history following diagnosis and treatment [16]. Several studies have utilized such ML models to detect CTS diagnosis as well as monitor progress. They utilize various types of data, such as MRI images, CT images, and clinical and historical data. For example, ref. [17] utilized a DL model to detect ultrasound (US) image features to make CTS diagnoses. The highest performance was 0.95%, with 0.94 in terms of accuracy and recall. Other studies [18,19] utilized a convolutional neural network (CNN) model to detect CTS diagnosis based on CT scan images. The developed model was validated using CT images aggregated from 53 patients and achieved an accuracy of 0.94%. The same model was used in [20,21,22,23], showing promising results. However, these studies have several limitations, including the following: (1) They depended on MRI and CT scan images, which are considered costly and may not be available for diagnosis. (2) The studies did not consider the overlap of CTS symptoms with other diseases, including cervical radiculopathysasas, de quervian tendinopathy, and peripheral neuropathy. (3) Several studies did not consider clinical, personal, and historical data, which have a significant effect in disease diagnosis as well as treatment. In this study, we utilized the ML model to perform CTS diagnosis based on patient historical, personal, and clinical examination data and US images and electrodiagnostics, and then predicted the probability of the patient improving [24,25,26].

### 1.3. Paper Contribution

The main contribution of our study can be summarized in the following points.

(1) Aggregate data about 80 CTS patients include 40 left hands and 40 right hands with different health status (mild, moderate, and severe) and 80 non-CTS patients with overlapping disease symptoms, including cervical radiculopathysasas, de quervian tendinopathy, and peripheral neuropathy.
(2) We build a Machine Learning model (bagging using random forest) that can distinguish between CTS and non-CTS patients based on BCTQ data and nerve conduction, and compare the results with several traditional machine learning models.
(3) We track and monitor the results after 1, 3, and 6 months through repeating the clinical examination tests and questionnaire.
(4) We build a machine learning model that predicts the probability of the patient improving after the hydro-dissection injection process based on the aggregated data after 3 different periods (1, 3, and 6 months).
(5) We use statistical tests such as ANOVA, *t*-test, and z-test to distinguish between patient status before and after the injection process and specify the features that have a significant impact on the probability of improvement.

### 1.4. Paper Organization

The rest of the paper is organized as follows. Section 2 details the literature review. Section 3 describes the data set that is used in the study. Section 4 clarifies the proposed work. The results and discussion are presented in Section 5. The paper concludes and future work is mentioned in Section 6.

## 2. Related Work

In this section, we discuss some recent research studies based on artificial intelligence to classify and diagnose CTS. Several attempts have been made to enhance the performance of CTS patients and to predict symptom improvement by utilizing machine learning and deep learning [27]. In Dougho Park et al. [28], seven ML algorithms (Neural Network (NN), Support Vector Machines (SVM), k-Nearest Neighbors (KNN), Classification And Regression Tree, Random Forest (RF), Stochastic Gradient Boosting (SGB), and eXtreme Gradient Boosting (XGB)) were estimated based on 1037 CTS hands with 11 variables each and were retroactively analyzed. In these datasets, CTS was corroborated, and its sharpness was categorized into mild, moderate, and severe by using electrodiagnosis in these datasets. XGB had the highest accuracy in multiclass classification, and its accuracy was 76.6% in test prediction as well as 76.1% during training. Konstantinos I. Tsamis et al. [29] used five machine learning techniques (Logistic Regression (LR), SVM, KNN, Decision Tree (DT), and Naive–Bayes (NB)) to examine the feasibility of automatically identifying median nerve mononeuropathy using conventional electrodiagnostic criteria utilized in clinical practice. Based on the NCS signal data analysis gathered from 38 volunteers, the outcomes of the classifiers were verified through neurophysiological and clinical diagnosis. With the most accurate classifier (SVM), automated classification between patients and controls achieved an accuracy of 0.9513 compared to NCS and 0.8906 compared to clinical diagnosis. The results show the ability of automated identification of carpal tunnel syndrome and can be employed in decision making, ultimately eliminating human error. US is the most popular imaging modality used to diagnose CTS to make up for the lack of nerve electrical inspection. Recently, using medical images, artificial intelligence algorithms have been used to accurately and human-error-free diagnose musculoskeletal diseases. For example, You-Wei Wang et al. [30] applied their study to 50 patients (8 healthy patients and 42 CTS patients), each having two cases (right and left hand). A Deep Similarity Learning (DeepSL) model known as MNT-DeepSL was proposed by the authors. The model’s formation was used to trace the problem of median nerve location on US images. The tracing of the median nerve is connected to image representation, input decision rules, a deep learning model called ResNet, and a different layer. The accuracy of the model’s performance was 0.9, which allows the physiatrist to locate the median nerve in the continuous US images. A deep and transfer learning system was developed by Issei Shinohara et al. [31] using US images to diagnose CTS accurately based on 60 cases (30 healthy patients and 42 CTS patients) Three DL models (ResNet50, MobileNet_v2, and EfficientNet) popularly used for medical image classification were selected for this study. The accuracy of ResNet-50 and MobileNet_v2 was the highest at 0.90, with a precision of 0.86, recall of 1.00 (all models), and 0.92 for F-measure. R.T. Festen et al. [32] rated two-dimensional median nerve characteristics and mobility accurately based on US images using a medium-sized dataset and U-Net model. Hafane et al. [33] combined a CNN with the probabilistic gradient vector flow (PGVF) method to identify the median nerve. CNN identifies the region of interest (ROI) around the nerve and a dataset collected from US images elicited from 10 videos. The results show a median Dice Similarity Coefficient (DSC) of 0.85. A prediction model was created and verified by Hoogendam L. et al. [20] for the likelihood of patient-reported symptom improvement 6 months following carpal tunnel release (CTR). One statistical algorithm and two machine learning algorithms (LR, RF, and gradient boosting machines) were taken into consideration for training predictors based on a cohort of 2119 patients who underwent a mini-open CTR and completed the BCTQ preoperatively and 6 months postoperatively [34]. In the validation data set, a gradient boosting machine with five predictors had the best calibration and discriminative performance (area under the curve). It had a satisfactory calibration, a sensitivity of 0.77, a specificity of 0.55, and an area under the curve of 0.723. The positive predictive value was 0.50, and the negative predictive value was 0.81. Bowman A et al. [35] used 35 neurons in the hidden layer, a sigmoidal transfer function, and conjugate gradient backpropagation with Powell-Beale restarts. The average AUC for this combination was 0.763 (95% CI 0.758–0.769).

Fariborz Faeghi et al. [12] proposed a diagnostic system for CTS based on radiomic features extracted from MNs in ultrasound images (so-called ultrasomal features). This is known as a Computer Aided Diagnostic (CAD) system, and the radiologists’ performance was evaluated. A CAD system can help radiologists accurately diagnose CTS. An SVM classifier was used, and an optimum accuracy of 90.1% was achieved. Haiying Zhou et al. [21] proposed a deep learning framework for carpal tunnel segmentation using MR images, known as Deep CTS. Deep CTS can effectively segment the CTS area and correct the intersection when combining the results. They applied their study to 333 CTS images and achieved an accuracy of 0.63. Conrad J. Harrison et al. [36] developed flowcharts with a machine learning technique entitled chi-squared automatic interaction detection, which could enable clinicians and patients to understand the chances of a patient improving with surgery. They also developed ML algorithms using QuickDASH response data from a regional database and achieved an accuracy of 0.72 and 0.76. A.A. Ardakani et al. [37] used a CTS support vector machine (SVM) and convolutional neural network (CNN). A total of 200 wristbands were included, including 100 CTS wristbands and 100 control wristbands. CNN had the best performance, with an ACC of 0.970, while SVM achieved an ACC of 0.925 in the testing dataset to diagnose CTS.

Despite their adequate performance, these studies have different limitations, which can be summarized as follows.

(1) the building of CTS classification models based on one type of data, which affects model performance.
(2) the non-considering of historical data’s impact on CTS classification.
(3) using a single model in CTS classification.
(4) the non-considering of patient health progression.

Therefore, in our study, we decided to combine historical data, medical examination data, and NCS and CSA data to provide a robust and accurate model, depending on an ensemble to effectively address various data.

## 3. Dataset Description 

### 3.1. Data Description 

#### 3.1.1. Dataset Collection

Between April 2019 and April 2020, the dataset was obtained retrospectively from the Neurology department at Kaferelshikh University Hospitals in Egypt. In all, 160 patients took part in this study, divided into two groups: (1) 80 patients diagnosed with CTS via ultrasound (US) guided median nerve hydro-dissection, and (2) 80 patients with other diseases that have an overlap in symptoms with CTS, such as cervical radiculopathysasas, de quervian tendinopathy, and peripheral neuropathy, from the Neurology department. The current study was submitted to the IRB at Kaferelshikh University’s Faculty of Medicine for approval. All patients were assured confidentiality and personal privacy throughout the trial. Patients felt free to leave the research at any time without consequence. The information acquired was not and will not be used for any other purpose.

#### 3.1.2. Study Cohorts

The dataset for CTS patients was aggregated using the following inclusion criteria: (I) patients between the ages of 20 and 60; (II) CTS manifestations; (III) NCS show delayed latency of the sensory or motor conduction of the median nerve; and (IV) patients did not respond to medical treatment at least three months after the onset of symptoms. Data from healthy normal volunteers was also gathered from Kaferelshikh university hospitals’ inpatient and outpatient clinics, which were matched for age and gender. Pregnant women, as well as those with thoracic outlet or brachial plexopathy, were excluded from the study. All of the patients volunteered to take part in the trial.

#### 3.1.3. Aggregated Features 

For each patient, the following data were aggregated:

Firstly, personal, and historical data: The patient’s historical data, including personal data (i.e., age, gender, BMI, occupation, marital status, special habits, family history of similar conditions, and previous surgical and medical problems).

Secondly, medical questionnaire: All patients were evaluated using a computerized CTS sheet, which included all characteristics from the BCTQ (Appendix A). The BCTQ is a patient-reported questionnaire that is used to assess the intensity of symptoms as well as the overall performance of the patient’s functions. It discusses the most-often-used tools for diagnosing and evaluating CTS. BCTQ has two models: symptom severity scale (SSS) and functional status scale (FSS). Both modules work independently and can be used either together or separately. 

SSS is an eleven-question test that evaluates a variety of criteria, including pain, paresthesia, numbness, weakness, nocturnal symptoms, tingling, and motor skill problems. FSS is an eight-question questionnaire that assesses the overlap between a patient’s symptoms and functions through questions on activities such as reading, bathing, carrying a grocery basket, holding a book, and so on. The BCTQ’s major strength is how quick, simple, and easy it is to administer. In our dataset, the questionnaire is repeated for all patients after one, three, and six months of treatment to record and track patient status. It should be noted that the score only considers the patient’s symptoms from the previous two weeks.

Patients’ medical records (pain, paresthesia, numbness, weakness, and nocturnal symptoms) and BCTQ functional status (writing, buttoning, holding, grasping, opening jars, household tasks, bathing, and dressing) as well as pre-intervention and post-intervention data at one, three, and six months were used.

**Figure 1:**
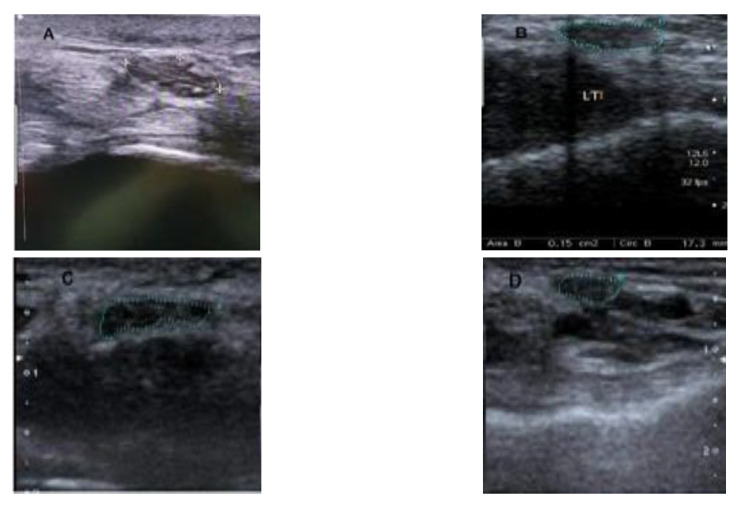
US examination of median nerve diameter (the dotted area) (**A**): pre-intervention, (**B**): at 1 month, (**C**): at 3 months, (**D**): at 6 months.

Thirdly, Ultrasonographic examination. A single sonographer is used to determine the CSA. It is calculated on the US machine using the tracing feature (in mm^2^ at the distal wrist crease), then directly traced around the inner border of the epineurium with no weaving between each fascicle [38]. This method was shown to be more accurate than the ellipsoid method. CTS is defined as a median nerve area more than 9 mm^2^. In this study, we followed the classifications of El Miedany et al. [39] of the CTS severity scale according to the CSA (up to 13.0 mm^2^ for mild, 13.0 to 15.0 mm^2^ for moderate, and more than 15.0 mm^2^ for severe) [2]. All measurements were taken three times, with the average result used for statistical analysis. Figure 1 shows the examination was repeated after 1, 3, and 6 months to track the progress of the treatment. All individuals in this trial received an injection of (1 mL lidocaine, 2 mL [8 mg]).

**Figure 2:**
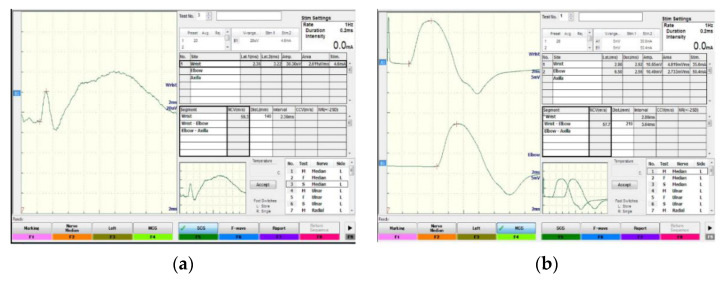
Nerve conduction for CTS patients (**a**) abnormal (**b**) normal.

Fourthly, NCS was performed by using an NCS Apparatus (NIHON KOHDIN), model MEB-9400K, Serial number SNI-00833, in the Neurology department, Kaferelshikh university hospitals. It was conducted for all patients in our study. Abnormalities on electrophysiological testing were considered the first criterion standard for CTS diagnosis. The cut-off point utilized in the NCS was the median nerve distal sensory latency of 3.5 milliseconds. Distal motor latency (DML) was increased by 90%, to >4.5 milliseconds onset latency [40]. Figure 2 shows normal ana abnormal of NCS for CTS and control subjects.

### 3.2. Data Preparation

#### 3.2.1. Outlier Detection

Outlier detection is the technique of identifying outliers among normal items. Performing outlier detection during data preparation is considered a critical step, since it has a significant impact on the classification and clustering models’ performance. Several techniques are utilized to solve the outlier problem; statistical techniques include proximity-based models, distance-based methods, etc. Despite the good performance of the above-mentioned methods, in this study we choose to depend on our medical expert to specify and resolve data outliers.

#### 3.2.2. Data Imputation

Missing values is a very common problem, especially with medical data. It could occur due to corruption or collection error. Missing values could negatively affect the classifier performance in terms of bias affect. Several basic methods are used to fill numeric values, such as mean, max, min and others, to fill categorical values such as the most frequent item [41]. In our study we have a small number of missing values in each column, ranging from (2–5) for each column. In order to gain a high accuracy in data after imputation, we choose a variable strategy for handling missing values, known as multivariate imputation by chained equations (MICE) [42,43]. This approach generates n full datasets by substituting n distinct values for missing variables. It then analyses n datasets and pools/combines them to produce a combined resulting dataset. This technique outperforms single imputation methods, but it requires more computational power.

#### 3.2.3. Data Scaling

The goal of data scaling methods for ML is to identify the areas of current machine learning where scalability plays a significant role and should be properly implemented to reduce uncertainty, incorrect results, or increases in cost/processing time. This method of data scaling changes the value of any feature’s smallest value to 0, while changing its largest value to 1 [44]. In this study, applied data scaling is based on the following equation:

(1) $$x\prime =\frac{x-\bar{x}}{\delta }$$

## 4. Proposed Work

### Proposed Machine Learning Model

The initial stage in bagging is to generate several models with different datasets based on the bootstrap sampling technique [45,46,47]. Each set of samples includes random samples from the original data. Each training sample was generated with the same size; however, some samples repeated in several training samples, while other samples appeared just in one training sample. Thus, if the original data set has N size, the size of each generated set also has the same size.

The second stage is to generate several models by applying the same model on the generated sample sets. In RF, we select random features to construct the optimum split.

**Figure 3:**
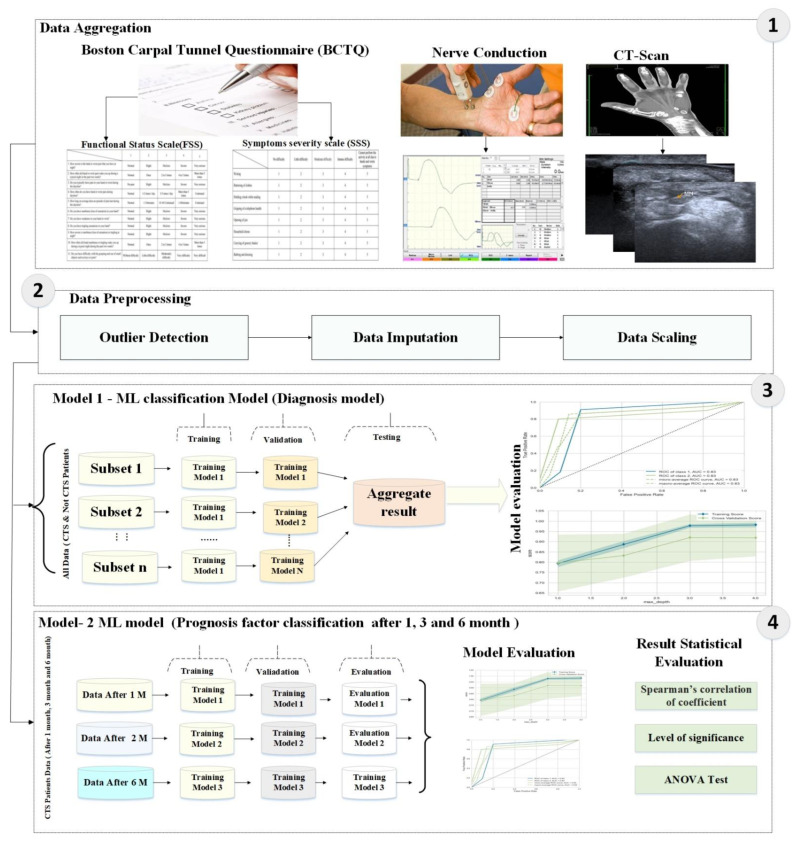
Proposed method architecture.

Unlike DT, which attempts to split based on the best feature to optimize errors, random forest involves random selection of features to construct the best split. On the other hand, when using bagging, DT always searches for the best feature for splitting. This leads to better correlation, where using bagging with different splitting of features leads to less correlation among subtrees. Therefore, using random forest with bagging results in constructing each tree with random samples, with each split built with a random sample of predictors. As shown in Figure 3 the proposed model is divided into four stages: (1) Data Aggregation, (2) Data prepossessing, (3) Build diagnose model, (4) Prognosis factor classification.

## 5. Results

This section is divided into two main sections: The first predicts CTS diagnosis based on several data sources in categories between CTS and non-CTS patients, and the second predicts the improvement of patient health status after the injection process after 1, 3, and 6 months.

### 5.1. Evaluation Metrics

The evaluation metrics of the CTS detection scenario include the following: accuracy, F1-score, sensitivity, specificity, and precision. These evaluation measures were computed using the TN (true negative), TP (true positive), FN (false negative), and FP (false positive) calculations. The number of correctly categorized negative and positive instances, respectively, is defined as TN and TP. The quantity of incorrectly identified positive and negative instances is also defined as FN and FP, respectively

(2) $$\mathrm{Recall}\;=\frac{\mathrm{TP}\;}{\;\left(\mathrm{TP}\;+\;\mathrm{FN}\right)}$$

(3) $$\mathrm{Specificity}\;\frac{\mathrm{TN}\;}{\left(\mathrm{FP}\;+\;\mathrm{TN}\right)}$$

(4) $$\mathrm{Precision}=\frac{\mathrm{TP}\;}{\left(\mathrm{TP}\;+\;\mathrm{FP}\right)\;}$$

(5) $$F1-\mathrm{score}\;=\frac{2\mathrm{TP}}{2T\;P\;+\;F\;P\;+\;F\;N}$$

(6) $$\mathrm{Accuracy}\;=\frac{TN+TP}{TP+FP+TN+FN}$$

### 5.2. Predicting CTS Diagnosis

**Figure 4:**
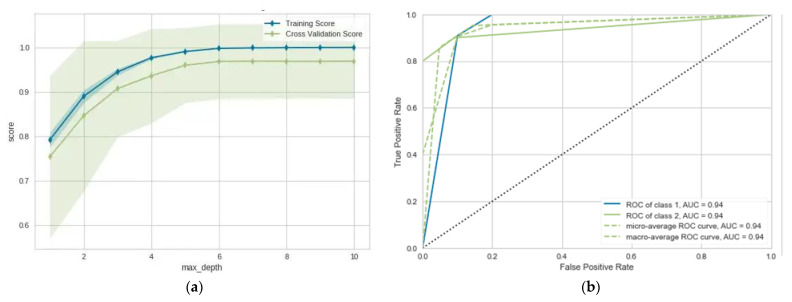
(**a**) Learning curve for the proposed model; (**b**) validation curve for proposed model (diagnosis model).

In this section, we use the data from the 160 patients included in this study. The patients were divided as follows: 80 patients diagnosed with CTS with different status (mild, moderate, and severe) and 80 patients without CTS, but having other diseases having an overlap with CTS. The total dataset was randomly divided into training and testing with percentages of 70% and 30%, respectively. As we can see in Table 1, the utilized models were able to differentiate between CTS and non-CTS patients with adequate performance ranging from 0.900% to 0.955%. As we can observe, LR and NB give the same performance: (ACC = 0.900, AUC = 0.924 for LR) and (ACC = 0.903, AUC = 0.921). MLP gives improved performance of about 3% in most evaluation metrics. The same applies for DT, where the proposed algorithm gives the best performance of (ACC = 0.955, AUC = 0.946). Predicting CTS prognosis will help differentiate between the CTS cases and other cases that may have similar symptoms. Figure 4 show the learning and validation curve for the proposed model.

### 5.3. Predicting Prognosis

#### 5.3.1. Predicting Prognosis after One Month

**Figure 5:**
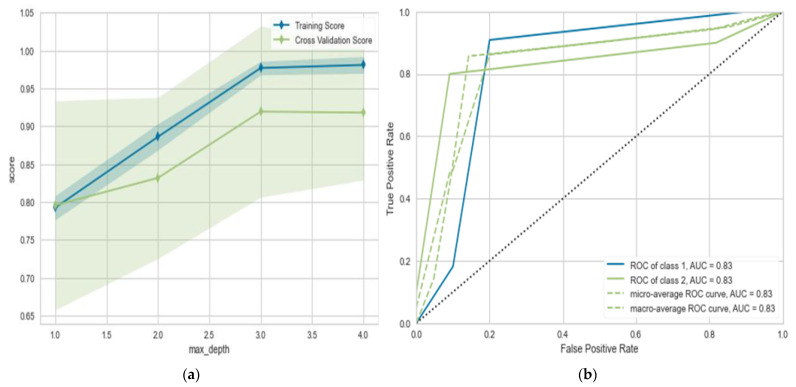
(**a**) Learning curve for the proposed model; (**b**) validation curve for proposed model (prognosis after 1 month).

In this section, we evaluate the performance of single classifiers from different types, including the linear model and statistical model, in predicting the prognosis after one month of medication. From Table 2, we can observe the following: (1) using LR gives the worst performance, achieving (*p* = 0.835%, R = 0.805%, F-measure = 0.824%, and ACC = 0.817%), followed by NB. (2) Using the SVC classifier gives adequate performance in terms of different evaluation metrics, achieving (*p* = 0.844%, R = 0.8159%, F-measure = 0.823%, and ACC = 0.833%). Using the MLP classifier gives the best performance in terms of traditional machine learning. It achieves metrics (*p* = 0.835%, R = 0.814%, F-measure = 0.814%, and ACC = 0.827%). The proposed algorithm improves the performance by 2–5% in terms of different evaluation metrics. It achieves (*p* = 0.875%, R = 0.876%, F-measure = 0.864%, and ACC = 0.877%). To visualize the performance of our proposed model. Figure 5 shows the learning and validation curve in both training and testing stages.

#### 5.3.2. Predicting Prognosis after Three Months 

**Figure 6:**
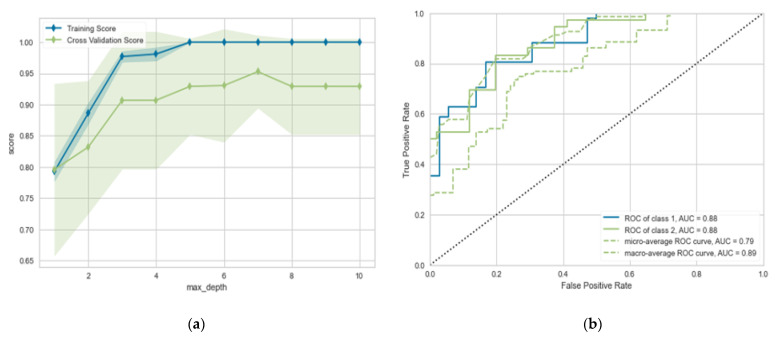
(**a**) Learning curve for the proposed model; (**b**) validation curve for proposed model (prognosis after 3 months).

In this section, we evaluate the performance of in predicting the prognosis after three months of medication. From Table 3, we can observe the following: (1) using LR gives the worst performance, achieving (*p* = 0.831%, R = 0.825%, F-measure = 0.831%, and ACC = 0.825%). (2) SVC and NB give similar performances; SVC achieved 0.831%, 0.820%, 0.838%, 0.818%, and 0.866% in terms of accuracy, P, R, and F measures, and AUC, respectively; the best performance obtained from DT with max depth = 6 according to different evaluation metrics, achieving (*p* = 0.855%, R = 0.852%, F-measure = 0.852%, and ACC = 0.900%). The proposed algorithm outperforms other algorithms by about 3–4%. It achieves (*p* = 0.911%, R = 0.900%, F-measure = 0.898%, and ACC = 0.901%). The model’s performance improved in predicting the prognosis in three months over predicting after one month by about 1–4% in most models. Figure 6 shows the learning and validation curve in both training and testing stages.

#### 5.3.3. Predicting Prognosis after Six Months

This section investigates the ability of our proposed ML model in predicting the prognosis of CTS after 6 months of medication. Table 4 shows the results of using traditional ML models as well as our proposed model. From that table, we can observe the following. (1) LR and NB give similar performance in terms of different metrics; NB achieved 0.821%, 0.813, and 0.823% and LR achieved 0.823%, 0.846%, and 0.8012% in terms of accuracy, precision, and recall, respectively. The best performance was obtained from the MLP classifier with one hidden layer according to different evaluation metrics, achieving (*p* = 0.85%, R = 0.833%, F-measure = 0.844%, and ACC = 0.832%). The proposed algorithm outperforms other algorithms by about 3–4% (*p* = 0.898%, R = 0.909%, F-measure = 0.898%, and ACC = 0.912%). The overall performance for predicting the prognosis after six months outperforms the prediction after one and three months.

**Figure 7:**
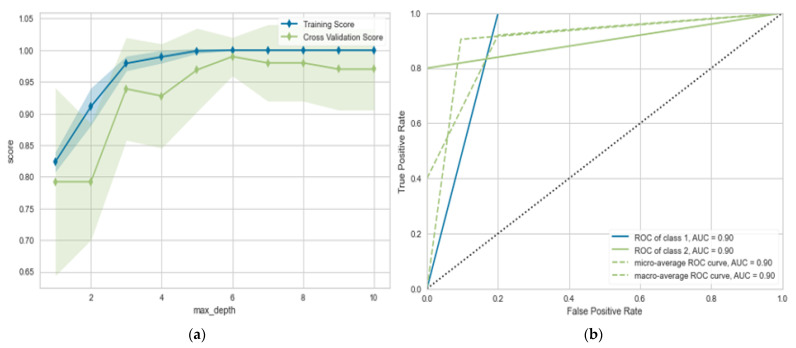
(**a**) Learning curve for the proposed model; (**b**) validation curve for proposed model (prognosis after 6 months).

The reason behind that improvement is based on several points, including (1) severity of the syndrome at presentation (decreased motor amplitude); (2) the extent of thenar muscle atrophy, and the patient’s primary employment (dentist, computer engineer, housewife, etc.); (3) using hormonal contraception via injection or oral means helps recurrence of entrapment. Obesity (BMI > 25), bilateral entrapment, hypothyroidism, uncontrolled diabetes, and uremia are additional systemic conditions that negatively affect the outcome of CTS treatment. Figure 7 shows the learning curve and the validation curve of the proposed model.

### 5.4. Comparison with Other Work

Carpal tunnel syndrome (CTS) is a clinical disease caused by compression of the median nerve in the carpal tunnel. Identifying the severity of CTS is essential to providing appropriate therapeutic interventions. Few studies have used a private small dataset that includes patient historical data, MRI images, US images, and CT images. Some studies used SVM to classify CTS patients. For example, ref. [28] utilized XGB with sample size 1073 [254(+), 761(−)] and achieved 0.76 in terms of ACC. In [29], the authors used SVM with a sample size of 64 [46(+), 19(−)] and achieved ACC = 0.9513, but the developed model may not be robust due to the small size of the dataset.

In [12], the authors used a sample size of 122 [65(+), 57(−)] and achieved the best performance of 0.901 using SVM. Though this study achieved adequate performance in terms of ACC, it showed inconsistency between true negative (TN) and true positive (TP) values.

Our proposed model utilized bagging with RF in 160 [80(+), 80(−)], achieving 0.955 and 0.946 in terms of ACC and AUC, respectively. Table 5 details the comparison with other studies in CTS classification. There are several reasons that our proposed model is superior to the state of the art: (1) Most studies have tried to categorize between CTS patients and normal patients, which may not make sense from the medical side. In our study, we aggregate the data from CTS patients and patients with other diseases that have an overlapping symptom with CTS, such as cervical radiculopathysasas, de quervian tendinopathy, and peripheral neuropathy. (2) Our proposed model considers historical data, which has a significant effect in disease diagnosis. (3) Our model did not show differences between training and testing and showed adequate consistency between TP and FP, which indicates the consistently of our model. Therefore, the proposed method could be considered as a generalized and robust model that could be used as an alternative to the clinical CTS diagnosis.

Table 6 shows the comparison between other studies in predicting prognosis. Very few studies are concerned with predicting improvement during the treatment process. All of these studies build their model on data after patient surgery. For example, [20] used gradient boosting to predict the probability of improvement after surgery based on the data aggregated from 2119 patients. They concluded with a model that could predict the progress with an AUC of 0.7229; in [36], the authors used XGB and achieved AUC = 0.791. We chose to make three models, to predict the improvement after one, three, and six months based on the data from questionnaire and the nerve condition. The proposed model achieved promising results in terms of different metrics for predicting after one month, (ACC = 0.875, AUC = 0.839), after three months (ACC = 0.901, AUC = 0.895), and after six months (ACC = V, AUC = 0.903). Accordingly, our model could be utilized to identify patients who may benefit from decompression. Our data-driven decision support tools can be used to help determine which patients to operate on, to avoid the associated risks and expenses of surgery.

### 5.5. Statistical Analysis

First, with regard to family history the statistical test shows that a median abnormality or prior carpal tunnel surgery had a substantial impact on CTS with a positive family history of the condition (chi square = 20.484, *p* < 0.001). Second, with regard to CSA with a cut-off point of 11 mm^2^, there was a statistically significant rise in CSA cases compared to controls. With a sensitivity of 95% and a specificity of 100%, this is regarded as a superb test for differentiating CTS patients from controls. Table 7 shows that there is a statistically significant interaction between the three US groups diagnosed with CTS (mild, moderate, and severe). Third, with regard to the questionnaire, we discovered a highly significant reduction in symptoms measured by the SSS, FSS, and pain analogue scale, as well as a reduction in CSA, after 1, 3, and 6 months post-injection when we assessed patient status and compared the baseline evaluation with the results after the injection process. Table 8 shows that, in terms of the medical questionnaire, there is a statistically significant interaction between the three groups of diagnosed CTS (mild, moderate, and severe).

## 6. Conclusions and Future Work

CTS can be differentiated easily from many other medical disorders based on ML; carpometacarpal arthritis of the thumb NCS shows normal values of terminal latency, conduction velocity, and amplitude. Dequervian neuropathy also shows normal parameter of NCS. Peripheral neuropathy shows abnormality within NCS according to the type of neuropathy: demyelinating or axonal neuropathy prolonged latency, decreased amplitude, and conduction velocity. Finally, the most common disorder confused with CTS is cervical radiculopathy, mainly C6 radiculopathy; this can also be easily differentiated by ML. In this study we develop the model as follows: First, we developed an ML model that could distinguish between CTS patients and non-CTS patients based on BCTQ data and nerve conduction and then compared the results with several machine learning models. Second, a prediction model was established for measuring the improvement as a result of the treatment process among CTS patients after one, three, and six months. The model was trained and validated using CTS patient data. The model displayed reasonable discriminative ability, high sensitivity, and reasonable specificity. In the future, we plan to extend our work to (1) try different hyper parameter tuning algorithms and compare the performance; (2) gather insights from different datasets to ensure comprehensiveness; (3) test our developed model using several real datasets to ensure robustness and generalization ability.

## Figures and Tables

**Table 1:** Results for CTS diagnosis.

Model	Training Score	Testing Score	Accuracy	Precision	Recall	F-Measure	AUC
LR	0.901 ± 0.001	0.900 ± 0.02	0.900 ± 0.001	0.915 ± 0.002	0.901 ± 0.012	0.901 ± 0.013	0.924 ± 0.010
NB	0.922 ± 0.011	0.902 ± 0.001	0.903 ± 0.001	0.930 ± 0.001	0.900 ± 0.012	0.917 ± 0.012	0.921 ± 0.010
SVC	0.931 ± 0.001	0.921 ± 0.011	0.920 ± 0.001	0.942 ± 0.013	0.911 ± 0.001	0.922 ± 0.002	0.933 ± 0.011
MLP	0.922 ± 0.001	0.921 ± 0.001	0.923 ± 0.011	0.931 ± 0.021	0.921 ± 0.011	0.961 ± 0.011	0.942 ± 0.010
DT	0.952 ± 0.012	0.931 ± 0.001	0.931 ± 0.002	0.940 ± 0.012	0.921 ± 0.001	0.931 ± 0.002	0.932 ± 0.001
Proposed	0.983 ± 0.011	0.955 ± 0.001	0.955 ± 0.001	0.963 ± 0.011	0.919 ± 0.012	0.933 ± 0.013	0.946 ± 0.010

**Table 2:** Results of the prognosis model after 1 month.

Model	Training Score	Testing Score	Accuracy	Precision	Recall	F-Measure	AUC
LR	0.828 ± 0.001	0.815 ± 0.01	0.817 ± 0.001	0.835 ± 0.002	0.805 ± 0.011	0.824 ± 0.013	0.819 ± 0.010
NB	0.861 ± 0.001	0.833 ± 0.011	0.828 ± 0.011	0.833 ± 0.012	0.833 ± 0.001	0.828 ± 0.016	0.828 ± 0.020
SVC	0.832 ± 0.001	0.821 ± 0.011	0.8331 ± 0.21	0.844 ± 0.013	0.8159 ± 0.01	0.823 ± 0.002	0.822 ± 0.011
MLP	0.842 ± 0.003	0.833 ± 0.001	0.827 ± 0.011	0.835 ± 0.021	0.814 ± 0.012	0.814 ± 0.019	0.801 ± 0.035
DT	0.886 ± 0.002	0.855 ± 0.001	0.853 ± 0.002	0.828 ± 0.012	0.859 ± 0.001	0.845 ± 0.002	0.821 ± 0.001
Proposed	0.916 ± 0.001	0.875 ± 0.01	0.877 ± 0.001	0.875 ± 0.002	0.876 ± 0.011	0.864 ± 0.013	0.839 ± 0.010

**Table 3:** Results of the prognosis model after 3 months.

Model	Training Score	Testing Score	Accuracy	Precision	Recall	F-Measure	AUC
LR	0.833 ± 0.002	0.821 ± 0.001	0.825 ± 0.001	0.831 ± 0.012	0.825 ± 0.002	0.831 ± 0.002	0.822 ± 0.001
NB	0.852 ±0.001	0.824 ± 0.023	0.821 ± 0.011	0.833 ± 0.001	0.823 ± 0.002	0.833 ± 0.002	0.846 ± 0.001
SVC	0.850 ±0.010	0.820 ± 0.001	0.831 ± 0.012	0.820 ± 0.002	0.838 ± 0.021	0.818 ± 0.001	0.866 ± 0.021
MLP	0.857 ± 0.001	0.85 ± 0.002	0.832 ± 0.003	0.851 ± 0.001	0.833 ± 0.002	0.844 ± 0.031	0.885 ± 0.013
DT	0.882 ± 0.003	0.863 ± 0.002	0.847 ± 0.021	0.855 ± 0.011	0.852 ± 0.002	0.852 ± 0.029	0.900 ± 0.015
Proposed	0.912 ± 0.0021	0.901 ± 0.001	0.901 ± 0.031	0.911 ± 0.022	0.900 ± 0.039	0.898 ± 0.011	0.895 ± 0.011

**Table 4:** Results of the prognosis model after 6 months.

Model	Training Score	Testing Score	Accuracy	Precision	Recall	F-Measure	AUC
LR	0.836 ± 0.002	0.825 ± 0.001	0.8231 ± 0.02	0.846 ± 0.012	0.8012 ± 0.002	0.825 ± 0.002	0.844 ± 0.001
NB	0.842 ±0.001	0.834 ± 0.023	0.821 ± 0.011	0.813 ± 0.001	0.823 ± 0.002	0.821 ± 0.002	0.835 ± 0.002
SVC	0.850 ±0.010	0.830 ± 0.001	0.831 ± 0.012	0.820 ± 0.002	0.818 ± 0.021	0.818 ± 0.001	0.856 ± 0.011
MLP	0.887 ± 0.001	0.85 ± 0.002	0.832 ± 0.003	0.85 ± 0.001	0.833 ± 0.002	0.844 ± 0.031	0.885 ± 0.033
DT	0.842 ± 0.003	0.842 ± 0.002	0.849 ± 0.011	0.825 ± 0.021	0.814 ± 0.002	0.824 ± 0.019	0.891 ± 0.035
Proposed	0.928 ± 0.0021	0.912 ± 0.001	0.912 ± 0.031	0.898 ± 0.022	0.909 ± 0.039	0.898 ± 0.011	0.903 ± 0.011

**Table 5:** Comparison with other work in terms of diagnosis detection.

Reference Year	Models	Dataset	Results	Type	Data Availability
[28]	XGB	761 CTS hands and 254 controls	ACC: 76.6	EDx	Public data
[29]	SVM	46 CTS hands and 19 controls	ACC: 0.9513	EDx	Provided upon request
[30]	MNT-DeepSL	84 CTS hands and 16 controls 16 controls	ACC: 0.90	US	Private
[12]	SVM	65 CTS hands and 57 controls	ACC: 0.901 AUC: 0.926	US EDx	Private
	Proposed	160 patients (80 CTS and 80 controls)	ACC = 0.955, AUC = 0.946	US, EDx BCTQ	Private

**Table 6:** Comparison with other work in terms of diagnosis prediction.

Reference	Models	Dataset	Results and Evaluations	Type	Medical Treatment
		1916 patients	ACC: 0.718, AUC: 0.791	BCTQ	Surgery
[36]	XGB	1916 patients	ACC: 0.718, AUC: 0.791	BCTQ	Surgery
[20]	Gradient boosting	2119 patients	AUC: 0.7229	BCTQ	Surgery
	Proposed	80 patients	ACC: 0.912, AUC: 0.903	BCTQ	Hydrodissection injection

**Table 7:** Changes in CSA after 1, 3, and 6 months.

Measurement	Mild	Moderate	Severe	*p* Value
Percentage	25%	30%	45%	<0.001
Median	1.1	0.7	0.4	
25th percentile–75th percentile	0.9–1.3	0.6–0.75	0.4–0.5	
Pairwise comparison	A	A	B	

**Table 8:** Descriptive statistics of FSS and SSS between the three US severity groups over time.

Parameter	Timing	Mild	Moderate	Severe	Group Time Interaction	
					F	*p*
FSS	Initial	24.6 ± 1.8	21.4 ± 2.8	24.8 ± 5.6	4.964	0.024
	One-Month	10.8 ± 1.3	10.8 ± 1.6	16.2 ± 2.2		
	Three-Month	12.6 ± 0.74	11.1 ± 1.3	17.8 ± 17.8		
	Six-Month	13.4 ± 0.94	12.4 ± 1.6	19.2 ± 19.1		
SSS	Initial	33.2 ± 3.4	33.8 ± 4.8	39.2 ± 5.1	9.112	<0.001
	One-Month	14.2 ± 2.1	17.2 ± 3.1	28.2 ± 6.6		
	Three-Month	18.2 ± 2.8	19.7 ± 2.7	30.2 ± 6.2		
	Six-Month	19.8 ± 2.8	21.5 ± 2.5	33.5 ± 6.2		

## Data Availability

Data available upon request.

## Abbreviations

The following abbreviations are used in this manuscript:

CTS	Carpal Tunnel Syndrome
NCS	Nerve Conduction Studies
SVM	Support Vector Machines
XGB	Extreme Gradient Boosting
LR	Logistic Regression
NB	Naive–Bayes
DT	Decision Trees
NN	Neural Network
KNN	k-Nearest Neighbors
RF	Random Forest
SGB	Stochastic Gradient Boosting
XGB	eXtreme Gradient Boosting
MLP	Multilayer Perception
US	Ultrasound
EDx	Electrodiagnostic
MNT	Median Nerve Localization
MRI	Magnetic Resonance Image
BCTQ	Boston Carpal Tunnel Syndrome Questionnaire
ROI	Region Of Interest
CNN	Convolutional Neural Networks
CSA	Cross-Sectional Area
SSS	Symptoms Severity Scale
FSS	Functional Status Scale
BMI	Body Mass Index

## Appendix A

Boston Carpal Tunnel Syndrome Questionnaire (BCTQ).

### Appendix A.1. Symptom Severity Scale (11 Items)

	**1**	**2**	**3**	**4**	**5**
1. How severe is the hand or wrist pain that you have at night?	Normal	Slight	Medium	Severe	Normal
2. How often did your hand or wrist pain wake you up during a typical night in the past two weeks?	Normal	Once	2 to 3 times	4 to 5 times	Normal
3. Do you typically have pain in your hand or wrist during the daytime?	No pain	Slight	Medium	Severe	No pain
4. How often do you have hand or wrist pain during the daytime?	Normal	1–2 times/day	3–5 times/day	More than 5 times	Normal
5. How long on average does an episode of pain last during the daytime?	Normal	<10 min	10~60 min Continuous	>60 min	Normal
6. Do you have numbness (loss of sensation) in your hand?	Normal	Slight	Medium	Severe	Normal
7. Do you have weakness in your hand or wrist?	Normal	Slight	Medium	Severe	Normal
8. Do you have tingling sensations in your hand?	Normal	Slight	Medium	Severe	Normal
9. How severe is the numbness (loss of sensation) or tingling at night?	Normal	Slight	Medium	Severe	Normal
10. How often did hand numbness or tingling wake you up during a typical night during the past two weeks?	Normal	Once	2 to 3 times	4 to 5 times	Normal
11. Do you have difficulty with the grasping and use of small objects such as keys or pens?	Without difficulty	Little difficulty	Moderately difficult	Very difficult	Without difficulty

### Appendix A.2. Functional Status Scale (8 Items)

	**No Difficulty**	**Little Difficulty**	**Moderate Difficulty**	**Intense Difficulty**	**Cannot Perform the** **Activity at All Due to** **Hand and Wrist** **Symptoms**
Writing	1	2	3	4	5
Buttoning of clothes	1	2	3	4	5
Holding a book while reading	1	2	3	4	5
Gripping of a telephone	1	2	3	4	5
Opening of jars	1	2	3	4	5
Household chores	1	2	3	4	5
Carrying of grocery basket	1	2	3	4	5
Bathing and dressing	1	2	3	4	5
